# A study on prescriptions contributing to the risk of high anticholinergic burden in adults with intellectual disabilities: retrospective record linkage study

**DOI:** 10.1186/s12991-022-00418-x

**Published:** 2022-10-29

**Authors:** McKernan Laura Ward, Sally-Ann Cooper, Angela Henderson, Bethany Stanley, Nicola Greenlaw, Christine Pacitti, Deborah Cairns

**Affiliations:** 1 Institute of Health and Wellbeing, University of Glasgow, Glasgow, G12 0XH Scotland; 2 Robertson Centre for Biostatistics, University of Glasgow, Glasgow, G12 8QW Scotland; 3 Greater Glasgow & Clyde NHS, Leverndale Hospital, Glasgow, G53 7TU Scotland

**Keywords:** Intellectual disabilities, Anticholinergic burden, Polypharmacy, Psychotropics

## Abstract

**Background:**

People with intellectual disabilities may face a disproportionate risk of experiencing high anticholinergic burden, and its negative sequalae, from a range of medications, and at younger ages than the general population, but there has been little previous study. Our aim was to determine the source of anticholinergic burden from prescribed medication.

**Methods:**

Retrospective matched observational study using record linkage. Adults with (*n* = 4,305), and without (*n* = 12,915), intellectual disabilities matched by age-, sex- and neighbourhood deprivation. The main outcome measure was the prescription of long-term (approximately 12 months use) anticholinergic medications overall (classified according to the Anticholinergic Risk Scale [ARS]), by drug class, individual drugs, and polypharmacy.

**Results:**

Adults with *n* = 1,654 (38.4%), and without *n* = 3,047 (23.6%), intellectual disabilities were prescribed medications long-term with anticholinergic effects. Of those on such drugs, adults with intellectual disabilities were most likely to be on central nervous system (62.6%), gastrointestinal (46.7%), and cardiovascular (28.4%) medications. They were prescribed more central nervous system, gynaecological/urinary tract, musculoskeletal, and respiratory medications, and less cardiovascular, infection, and endocrine medications than their matched comparators. Regardless of age, sex, or neighbourhood deprivation, adults with intellectual disabilities had greater odds of being prescribed antipsychotics (OR = 5.37 [4.40–6.57], *p* < 0.001), antiepileptics (OR = 2.57 [2.22–2.99], *p* < 0.001), and anxiolytics/hypnotics (OR = 1.28 [1.06–1.56], *p* = 0.012). Compared to the general population, adults with intellectual disabilities were more likely to be exposed to overall anticholinergic polypharmacy (OR = 1.48 [1.33–1.66], *p* < 0.001), and to psychotropic polypharmacy (OR = 2.79 [2.41–3.23], *p* < 0.001).

**Conclusions:**

Adults with intellectual disabilities are exposed to a greater risk of having very high anticholinergic burden through polypharmacy from several classes of medications, which may be prescribed by several different prescribers. There is a need for evidence-based recommendations specifically about people with intellectual disabilities with multiple physical and mental ill-health conditions to optimise medication use, reduce inappropriate prescribing and adverse anticholinergic effects.

**Supplementary Information:**

The online version contains supplementary material available at 10.1186/s12991-022-00418-x.

## Background

Adults with intellectual disabilities experience an increased risk of adverse effects associated with high anticholinergic burden [1–4]. Previous literature has focused predominantly on the older adult general population and found high anticholinergic burden to be associated with a range of poor health outcomes including cognitive impairment and dementia [5]; emergency health service use [6], and all-cause mortality [7–9]. Common adverse effects of anticholinergic burden include constipation, dry mouth, blurred vision, cognitive impairment, urinary retention, and increased fall risk. Some recent studies have also included younger adults. A UK Biobank study investigating 502,538 healthy adults found high anticholinergic burden in 27–73 year olds was modestly associated with cardiovascular events, hospital admissions (due to fall/fractures, and dementia/delirium) and mortality [9]. The cumulative impact of anticholinergic burden is, therefore, a pertinent issue in the pharmacotherapy of all patients across the lifespan.

People with intellectual disabilities have poorer health than the general population, and often have long-term health problems. Higher multimorbidity and more complex health needs occur at younger ages and continue throughout the lifespan, with poorer health outcomes and premature mortality [10]. Subsequently, people with intellectual disabilities are more likely to experience polypharmacy (taking multiple medication concomitantly, e.g., 5 +) [11] with complex pharmacotherapy, that can result in a significantly increased risk of adverse effects. The mean number of physical health conditions experienced by adults with intellectual disabilities is 11.04 [12], as well as 40% experiencing mental ill-health [10]. Unsurprisingly, one study reported that for adults with intellectual disabilities living in rural areas, in addition to a median of 8 (IQ = 4–11) contacts with primary health care over a year, 43.6% also had contacts with secondary or tertiary health care, 10.3% had hospital admissions, 10.3% had emergency GP contacts, and 20.5% presented to accident and emergency departments [13]. To a lesser extent, the figures were also high for participants in urban areas [13]. Hence, many adults with intellectual disabilities are treated by multiple prescribers. Moreover, prescriptions initiated in hospital settings are managed in the community, and many general practitioners in primary care, report being concerned changing anticholinergic medications initiated by specialists [14].

Whilst some increased medication use is appropriate, given the higher prevalence of physical and mental ill-health, there are an increasing number of guidelines and initiatives aimed at reducing inappropriate prescribing, considering medication review and de-prescribing where appropriate [15–17]. Previous evidence has indicated psychotropics to be the main drug class responsible for high anticholinergic burden in older adults with intellectual disabilities (aged 40 +) [3]. Within this class of medications, a large focus of attention has been on the off-label use of antipsychotics to manage ‘challenging behaviour’ in this population [18]. This is despite high anticholinergic risk, lack of substantial evidence base of effectiveness in this context, and no clear indication for antipsychotic use [18]. If there is no diagnosis of mental ill-health, best practice guidelines recommend short-term use only with review of efficacy after 3–4 weeks [16], and as part of a multimodal treatment approach, i.e., non-pharmacological psychosocial or behavioural interventions [19].

This paper reports on the contribution of different classes of drugs to the risk of high anticholinergic burden in adults with intellectual disabilities compared to those in the general population, and also investigates psychotropic prescriptions in further detail.

## Methods

### Study cohort

The research was approved by Scotland’s Public Benefit and Privacy Panel for Health and Social Care (reference 1516–0281). The National Health Service (NHS) Greater Glasgow and Clyde Primary Care Intellectual Disabilities Register of 2014 was created from multiple sources (e.g., general practitioners who were financially incentivised to identify all their patients with intellectual disabilities (100% did so) and community intellectual disabilities teams) and updated annually thereafter. It was used to identify all adults (aged 17 +) with intellectual disabilities within the defined geographical boundary of NHS Greater Glasgow and Clyde, which forms almost a quarter of the Scottish population. Adults with intellectual disabilities were each matched to three general population adults based on age (year of birth), sex, and neighbourhood deprivation (Scottish Index of Multiple Deprivation: SIMD2016 using the postcode area). Whilst health in the general population is closely linked to neighbourhood deprivation [20], previous research suggests this gradient is not evident in the intellectual disabilities population [21]. However, people with intellectual disabilities are more likely to live in the more deprived areas. This distinction is important, as public health initiatives designed for the general population that are focussed on more deprived areas build in disadvantage to people with intellectual disabilities living in more affluent areas, but who have health inequalities. Therefore, we chose to match the groups to reflect the difference structure of the intellectual disabilities population compared to the general population. In Scotland, patient health records are identified using their unique patient identifier; the Community Health Index (CHI), which is held centrally by National Services Scotland (NSS). The CHI database was used for this matching and linkage process. NSS also holds the Prescribing Information System database, which was used to identify 1 year of anticholinergic medication prescriptions between September 2016 and August 2017 for the study groups. This data source records all community prescribed and dispensed medications in Scotland and classifies them via British National Formulary (BNF) categories. NHS Scotland’s Information Services Division completed data linkage and extraction in August 2017. The study group consisted of 17,228 but during data cleaning the research team identified two general population adults as having intellectual disabilities. Since each adult with intellectual disabilities was matched with general population adults 1:3 to create one ‘cluster’, all participants from these matched clusters were excluded (*n* = 8), leaving a sample size of *n* = 17,220. Further details on sampling and linkage have previously been reported [4].

Anticholinergic medications were identified using the modified Anticholinergic Risk Scale (ARS) which was updated by the authors [4]. The ARS list classifies anticholinergic medicines as moderate (risk category 1), strong (risk category 2), and very strong (risk category 3), and is one of the more conservative anticholinergic burden scales [9]. There are numerous anticholinergic burden scales, although there is consensus of a cumulative total score of 3 or greater to be clinically at risk of adverse effects. To determine the main contributors to anticholinergic burden, prescriptions were only included if there were 3 or more repeat prescriptions constituting approximately 9–12 months usage during the year being studied. Anticholinergic drugs were those listed in the updated ARS and categorised according to the BNF chapter, and psychotropic sub-chapters.

### Statistical analysis

Data were summarised descriptively by group (those with, and without intellectual disabilities), for each medication outcome. Outcomes investigated were individual medication classes, psychotropic prescribing (antipsychotics, antiepileptics, antidepressants, anxiolytics/hypnotics) and polypharmacy. Group comparisons were made using mixed-effects binary logistic regression models with a fixed effect for group, and a random effect for the matched cluster. Group effects are reported from models adjusted for known confounders (sex, age category, SIMD quintile), with the general population as reference and intellectual disabilities cohort as effect level.

Interaction terms between group and each main effect (sex, age, SIMD quintile) were added to adjusted models and when significant, explored using both odds ratios (OR) and marginal effects at representative values (MERs). OR [95% Confidence Intervals] and *p* values are reported for the adjusted group effect tested within each level of the main effect, e.g., significant interaction between group (adults with and without intellectual disabilities) and age examined by modelling the group effects within each age category. Using the full model (main effects and interaction term), the MER analyses shows the magnitude of effects allowing us to obtain predicted probabilities of the group effect on each outcome. Adjusted MERs describe the strength of an association between a risk factor (e.g., group) and the outcome (e.g., antipsychotic use), allowing us to see how the marginal effect differs across that range (e.g., change across age categories), at representative values of covariates (held constant) [22], e.g., the predicted probability of being on an antipsychotic if an adult is in the intellectual disabilities group and aged 55–64 (within group analysis). Values were chosen on the basis of no statistically significant effects of that covariate in the group interaction term (e.g., average of sex and SIMD for group differences in antipsychotic prescribing). Statistical analyses were conducted in Stata (StataCorp 2017).

## Results

### Demographics

The full data set included *n* = 4,305 adults with intellectual disabilities and *n* = 12,915 general population adults. General demographics for this full sample can be seen in Table 1. Of the full sample, there were 1,654 (38.4%) adults with intellectual disabilities prescribed long-term anticholinergic medication, compared to 3,047 (23.6%) adults without intellectual disabilities. These groups comprise our population for all analyses (Table 1).

**Table 1:** Descriptive statistics for total sample and subgroup of those prescribed long-term anticholinergic medication (≥ 3 repeat prescriptions over 12 months)

Characteristic	Total		AC medications	
	ID	GPop	ID	GPop
Population	4,305	12,915	1,654	3,047
Sex				
Male	2,504 (58.2%)	7,512 (58.2%)	925 (55.9%)	1,628 (53.4%)
Female	1,801 (41.8%)	5,403 (41.8%)	729 (44.1%)	1,419 (46.6%)
Age categories				
17–24	403 (9.4%)	1,209 (9.4%)	96 (5.8%)	38 (1.3%)
25–34	811 (18.8%)	2,433 (18.8%)	224 (13.5%)	172 (5.6%)
35–44	673 (15.6%)	2,019 (15.6%)	210 (12.7%)	282 (9.3%)
45–54	965 (22.4%)	2,895 (22.4%)	418 (25.3%)	726 (23.8%)
55–64	794 (18.4%)	2,382 (18.4%)	366 (22.1%)	859 (28.2%)
65–74	464 (10.8%)	1,392 (10.8%)	235 (14.2%)	645 (21.2%)
75 +	195 (4.5%)	585 (4.5%)	105 (6.4%)	325 (10.7%)
SIMD				
1-most deprived	2,293 (53.3%)	6,879 (53.3%)	867 (52.4%)	1,821 (59.8%)
2	848 (19.7%)	2,544 (19.7%)	331 (20.0%)	605 (19.9%)
3	545 (12.7%)	1,635 (12.7%)	233 (14.1%)	335 (11.0%)
4	349 (8.1%)	1,047 (8.1%)	135 (8.2%)	173 (5.7%)
5-least deprived	270 (6.3%)	810 (6.3%)	88 (5.3%)	113 (3.7%)

*AC* anticholinergic, *GPop* general population, *ID* intellectual disabilities

### Anticholinergic drugs across medication classes

For the adults with intellectual disabilities prescribed anticholinergic medication, these were most likely to be central nervous system (62.6%), gastrointestinal (46.7%), cardiovascular (28.4%), and respiratory system medications (13.4%) (Table 2). The pattern differed for the general population (though may not be representative of the whole general population who are older, have equal sex ratio, and a different distribution of SIMD, due to the matching with the intellectual disabilities group). Compared to the matched general population, the adults with intellectual disabilities were more likely to be prescribed central nervous system (2.23 [1.96–2.53]), obstetrics, gynaecology, urinary tract (2.82 [1.83–4.34]), and musculoskeletal (2.49 [1.69–3.67]) medications and were less likely to be prescribed cardiovascular (0.61 [0.53–0.71]), infections (0.43 [0.21–0.68]), and endocrine (0.45 [0.29–0.68]) medications.

**Table 2:** Number and percentage of adults with intellectual disabilities prescribed anticholinergic medication by medication class (BNF chapter), in comparison with general population adults

BNF chapter	Adults prescribed anticholinergic medications (*n* = 4,701)		OR [95% CI], *p* value*
	Intellectual disabilities (*n* = 1,654)	General population (*n* = 3,047)	
Gastro-intestinal system	773 (46.7%)	1,505 (49.4%)	1.00 [0.88–1.13], *p* = 0.985
Cardiovascular system	477 (28.4%)	1,419 (46.6%)	0.61 [0.53–0.71], *p* < 0.001
Respiratory system	222 (13.4%)	373 (12.3%)	1.06 [0.88–1.27], *p* = 0.547
Central nervous system	1,037 (62.6%)	1,231(40.4%)	2.23 [1.96–2.53], *p* < 0001
Infections	9 (0.54%)	53(1.7%)	0.43 [0.21–0.86], *p* = 0.017
Endocrine system	29 (1.8%)	120 (3.9%)	0.45 [0.29–0.68], *p* < 0.001
Obstetrics, gynaecology and urinary-tract disorders	51 (3.1%)	40 (1.3%)	2.82 [1.83–4.34], *p* < 0.001
Musculoskeletal and joint diseases	65 (3.9%)	40 (1.3%)	2.49 [1.69–3.67], *p* < 0.001

All regression models are adjusted for sex, age, and SIMD (neighbourhood deprivation)

^*^OR Odds Ratio, 95% *CI* Confidence Interval and corresponding *p* value for the Intellectual Disabilities group effect with the General Population as the reference group

Table 3 shows the most commonly prescribed anticholinergic drugs (ranked 1–10) for the intellectual disabilities group; 5 are psychotropics, 3 are cardiovascular, and the remaining 2 are gastrointestinal.

**Table 3:** Top 10 anticholinergic medicines prescribed in adults with intellectual disabilities compared to general population controls

Medication (ARS risk category)	ID (*n* = 1,654)	Rank	GPop (*n* = 3,047)	Rank
Omeprazole (1)	26.5%	1	29.0%	1
Carbamazepine (1)	16.4%	2	1.5%	29
Lansoprazole (1)	12.9%	3	12.6%	4
Ramipril (1)	11.9%	4	15.3%	3
Lamotrigine (1)	10.6%	5	1.1%	34
Risperidone (1)	10.2%	6	0.9%	38
Amlodipine (1)	9.6%	7	17.1%	2
Sertraline (2)	9.0%	8	9.3%	6
Diazepam (2)	7.9%	9	5.7%	11
Bendroflumethiazide (1)	6.2%	10	9.9%	5

Percentages are presented from the subgroup sample taking long-term anticholinergic medicines

*ARS* Anticholinergic Risk Scale, *ID* Intellectual Disabilities, *GPop* General Population

### Psychotropic prescribing

Of the anticholinergic central nervous system drugs that were prescribed, the majority (> 99%) were psychotropics (antipsychotics, antiepileptics, antidepressants, anxiolytics/hypnotics). Compared to the adults without intellectual disabilities, regardless of sex, age or neighbourhood deprivation, adults with intellectual disabilities had greater odds of being prescribed antipsychotics (5.37 [4.40–6.57]); antiepileptics (2.57 [2.22–2.99]); and anxiolytics/hypnotics (1.28 [1.06–1.56]), all statistically significant at *p* < 0.01. The likelihood of anticholinergic antidepressant prescribing was similar between the groups (*p* = 0.174). Table 4 shows the number and percentage of adults with, and without, intellectual disabilities prescribed psychotropic drugs for each of these subcategories.

**Table 4:** Number and percentage of adults with intellectual disabilities prescribed anticholinergic psychotropic medication by subcategory, in comparison with general population adults

Psychotropic subcategory	Intellectual disabilities (*n* = 1,654)	General population (*n* = 3,047)	OR [95% CI], *p* value*
Antipsychotics	411 (24.9%)	173 (5.7%)	5.37 [4.40–6.57], p < 0.001
Antiepileptics	486 (29.4%)	393 (12.9%)	2.57 [2.22–2.99], p < 0.001
Antidepressants	206 (12.5%)	383 (12.6%)	0.87 [0.71–1.06], p = 0.174
Anxiolytics/hypnotics	222 (13.4%)	289 (9.5%)	1.28 [1.06–1.56], p = 0.012

Numbers and percentages are presented from the subgroup sample taking long-term anticholinergic medicines. All regression models are adjusted for sex, age, and SIMD

^*^OR Odds Ratio, 95% *CI* Confidence Interval and corresponding *p* value for the Intellectual Disabilities group effect with the General Population as the reference group

**Fig. 1:**
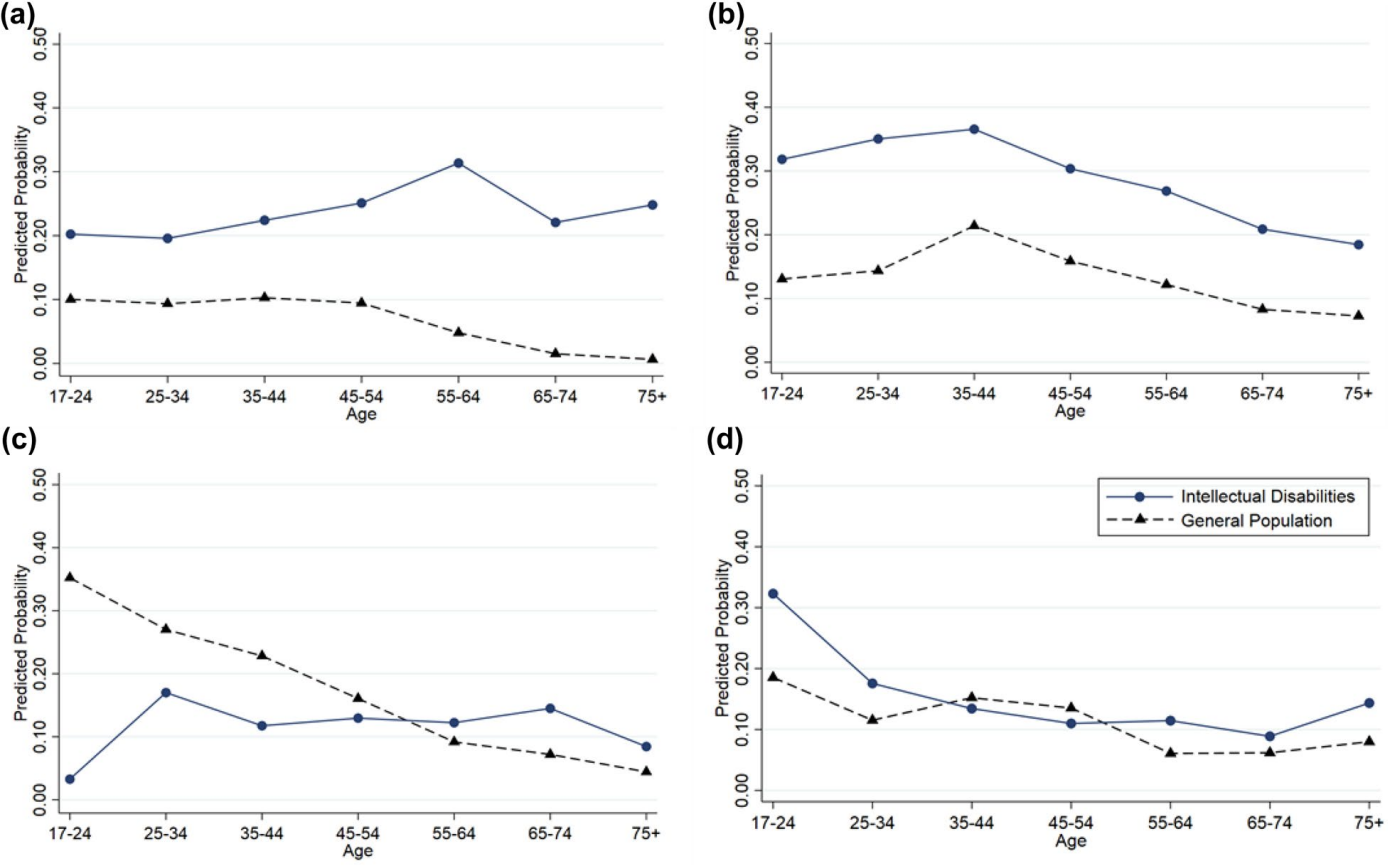
Influence of age and groups’ MERs for predicted probability of being on **a** antipsychotics, **b** antiepileptics, **c** antidepressants, and **d** anxiolytics/hypnotics

Extending these findings to include the interaction terms between group and the main effects (sex, age, neighbourhood deprivation), revealed some statistically significant results. The interaction between group and age was significant for antipsychotics (*p* < 0.001), antidepressants (*p* < 0.001) and anxiolytics/hypnotics (*p* = 0.01), whilst the interaction between group and neighbourhood deprivation was significant for antiepileptics (*p* < 0.001). ORs for the subgroup analyses by age (Additional file 1: Table S1) and deprivation (Additional file 1: Table S2) are reported. Figure 1 plots the MERs by group and age, averaging across sex and SIMD, demonstrating the change in risk factors that affect the probability that the individual is on a specific type of psychotropic (1 representing a 100% likelihood).

Prescribing of antipsychotics in the general population decreased from younger to older adults, but for adults with intellectual disabilities it peaked at 55–64 years, hence the extent of the difference between the groups increased with age (Fig. 1a). Antipsychotic statistically significant group differences start from age group 25–34 (2.48 [1.34–4.60]), rising over each age group, to age 75 + (61.07 [13.11–284.44]) (Additional File 1: Table S1). Age-related prescribing patterns of antidepressant and anxiolytic/hypnotics showed fewer statistically significant results. Whereas the general population show a decrease in antidepressant prescriptions from younger to older adults, the intellectual disabilities group fluctuated; ORs were significantly lower for the intellectual disabilities group at ages 17–24 (0.06 [0.01–0.27]), and higher for ages 65–74 (2.21 [1.35–3.64]) (Fig. 1b, Additional File 1: Table S1). Differences in anxiolytic prescribing between the groups were statistically significant, with increased prescribing for the intellectual disabilities group for 17–24 years (2.76 [1.00–7.63]) and 55–64 years (1.97 [1.28–3.03]) but did not differ at other ages (Additional File 1: Table S1). With regard to antiepileptics, the extent of differences between groups showed a positive gradient across SIMD, with the smallest difference in the most deprived neighbourhood (1.94 [1.59–2.37]), and the greatest difference being in more affluent areas (8.77 [3.64–21.15]) (Additional File 1: Table S2 and Figure S1). This was due to higher prescriptions of antiepileptics in more deprived neighbourhoods in the general population, but not in the adults with intellectual disabilities.

### Polypharmacy

The range of different anticholinergic medicines a person was prescribed was between 1 and 11 for both groups, with a median of 2. Anticholinergic polypharmacy (concurrent use of 2 + anticholinergic medications) was significantly higher in the adults with intellectual disabilities group with 61.3% (*n* = 1,013) prevalence compared to 54.7% (*n* = 1,668) (OR = 1.61 [1.41–1.83], *p* < 0.001). Psychotropic polypharmacy (concurrent use of 2 + psychotropic medications) was also significantly higher in the adults with intellectual disabilities group with 33.7% (*n* = 558) prevalence compared to 14.3% (*n* = 435) (OR = 2.79 [2.41–3.23], *p* < 0.001). These results are adjusted for age, sex, and neighbourhood deprivation. Adults with intellectual disabilities are almost three times more likely to be exposed to psychotropic polypharmacy compared to the general population.

## Discussion

### Principal findings and interpretation

Our findings are important, as they demonstrate that the high rates of anticholinergic burden experienced by adults with intellectual disabilities at all ages (not just older ages) are due to several classes of medications, not only psychotropic, and which may be prescribed by several different clinicians. Whilst the general practitioner has an overview, it is important for all clinicians to be cognisant of this issue. Of the 38.4% of adults with intellectual disabilities on long-term anticholinergic prescriptions, the medications most frequently prescribed were central nervous system medications (62.6%), gastro-intestinal medications (46.7%), and cardiovascular medications (28.4%), and polypharmacy (both anticholinergic and psychotropic) was more common than monotherapy.

Examining psychotropic contributions to anticholinergic burden showed that adults with intellectual disabilities were more likely to be prescribed antipsychotics, antiepileptics and anxiolytics/hypnotics compared to adults without intellectual disabilities, and had similar levels of antidepressant prescriptions. Results on antipsychotic medication use support previous evidence of an age-related increase for those with intellectual disabilities [18, 23–25], in contrast to the general population who showed a decreased use across age (Fig. 1). Whilst we find statistically significant differences between the groups from ages 25 onwards, the smaller numbers in older age groups incur large confidence intervals (Additional File 1: Table S1). The current results and previous evidence [18, 26], indicate long-term antipsychotic use, despite associated health risks, including anticholinergic effects, and best practice guidelines advocating non-pharmacological approaches in the long-term [16]. These findings suggest antipsychotics comprise a significant contribution to high anticholinergic burden.

Regarding anticholinergic antiepileptics, the greatest group difference in the most affluent neighbourhoods (Additional File 1: Table S2, Figure S1). This reflects the higher rates of prescribing in more deprived areas in the general population, which is a typical finding [10]. Prescribing is high across all neighbourhoods for the intellectual disabilities population, with no obvious deprivation gradient. This highlights the need for medication reviews and service provision in all neighbourhoods regardless of extent of deprivation.

### Polypharmacy

Anticholinergic polypharmacy across all drug classes was present, and at 1.6 greater odds for the adults with intellectual disabilities. The combination of multiple lower risk drugs (ARS score 1) and high polypharmacy in adults with intellectual disabilities contributes to the overall clinical risk of high anticholinergic burden. Non-anticholinergic licensed alternatives could be explored in place of the more commonly used anticholinergic medicines. A realistic medicine patient centred approach is key and the prescription of psychotropic medication should be “informed by a comprehensive biopsychosocial assessment” [19]. Rates of multimorbidity (physical and mental ill-health) are high in people with intellectual disabilities [10], so polypharmacy (including psychotropic) can be indicated and appropriate. For example, around 25% of adults with intellectual disabilities have epilepsy requiring antiepileptic medications [12]. Although, antiepileptic drugs are commonly prescribed as mood-stabilisers and not only seizure-control [27], which can increase the risk of high anticholinergic burden.

Clinical services are typically organised around single conditions, which can pose additional risks for polypharmacy. Comprehensive, regular, and targeted medication reviews would help to identify excess prescribing. To see where medications can be rationalised one drug at a time, to establish support for treatment adherence, to enable good communication between the person’s different health care professionals/teams if they are accessing multiple services, and not focus simply on single-disease care pathways. This is well-recognised in the general population, e.g., recent NICE clinical guideline on multimorbidity [28], but has received little attention for people with intellectual disabilities. For example, a review on the effectiveness of medication reviews for those with intellectual disabilities included only 8 studies, with just 3 of good quality; all of which evidenced the reduction in medication-related problems after multidisciplinary medication reviews [29].

### Comparison with previous literature

Inappropriate polypharmacy is a problem among adults with intellectual disabilities and has a high risk of adverse effects. Previous evidence from representative population samples of adults with intellectual disabilities report a high prevalence of medicine use (average of 4–7 prescriptions) and polypharmacy (overall between 21% and 38%, psychotropic polypharmacy between 23% and 41%) [30–33]. Higher rates of polypharmacy (54%) and psychotropic polypharmacy (66%) have been reported in older adults with intellectual disabilities (aged 40 +) [2, 34]. Our data are comparable to these results; similar to Axmon et al. [1], we find that most adults take at least 2 anticholinergic medications irrespective of age or sex. Our data include only anticholinergic prescriptions, therefore, are likely an underestimation of overall prescriptions and polypharmacy, e.g., many adults with intellectual disabilities take the antiepileptic sodium valproate, which is psychotropic but not anticholinergic. Results show adults with intellectual disabilities were 2–3 times more likely to experience long-term polypharmacy compared to their peers. Our findings support previous work reporting high psychotropic polypharmacy in this patient population to be predominantly due to antipsychotic and antiepileptic medication use. [3, 24]

Polypharmacy and anticholinergic burden are inter-related, but both show an independent dose–response relationship with all-cause hospital admissions and mortality in general population adults [9, 35]. Polypharmacy prevalence is increasing; a recent UK Biobank study reported anticholinergic burden to be 3–9 times higher between 1990 and 2015 [36]. Inappropriate prescriptions pose a health risk, adults with intellectual disabilities have a 2.7 times greater odds of hospitalisation due to psychotropic adverse medication events [37]. More generally, there is evidence that every prescribed drug leads to an increase in having a potential drug–drug interaction of clinical significance for this population (OR = 0.87 [0.72–1.00]) [31]. People with intellectual disabilities often experience multimorbidity which may necessitate polypharmacy, but this can also mean increased exposure to potential drug–drug interactions. Moreover, both polypharmacy and multimorbidity were independent significant predictors for mortality in older (aged 50 +) adults with intellectual disabilities [38]. The precise relationship between polypharmacy, anticholinergic burden, multimorbidity, and mortality is yet to be clarified.

### Strengths and limitations

The strength of the current study lies in the sample; ours was a large representative group of adults with intellectual disabilities age-, sex-, and neighbourhood deprivation-matched to general population adults without intellectual disabilities within Scotland’s largest NHS health board. The use of this record linkage allows for robust information on prescribed medication with anticholinergic effects, although only a year of data was analysed. The study’s specific focus is on anticholinergic medication, and not the total rates of encashed prescriptions; so conclusions on overall prescribing cannot be drawn. In addition, as this is prescription data, there is no clarity of patient usage, dosage, or clinical indication. Therefore, an important limitation is the lack of clinical data on the conditions, diseases, or indeed, actual anticholinergic burden as measured by serum activity assays. There is also a lack of information on the severity of the intellectual disabilities. Finally, we were unable to distinguish between regular or pro re nata (PRN) medication in our database. Whilst our conservative definition of only including medications with 3 + repeat prescriptions over the 12 months is highly likely to have included only long-term anticholinergic medication use, we cannot say with 100% certainty that it did not include some people with very high use of PRN (as required) medication. In addition, this means that we may have underestimated the extent of exposure to anticholinergic medication in view of the use of PRN antipsychotics in this population which did not meet our threshold definition.

## Conclusions

In line with evidence from the general population, our data clearly show that drugs with a moderate anticholinergic risk from different drug classes contribute to the risk of clinically high anticholinergic burden [39]. In several cases, prescriptions of these drugs are unavoidable, for example, to manage complex epilepsies, however, given the multiple prescribers, an overview review by a clinician identified as responsible is essential to reduce or discontinue drugs when no longer needed, and avoid unnecessary repeat prescriptions. People with intellectual disabilities are particularly vulnerable, with commonplace psychotropic polypharmacy as well as overall anticholinergic polypharmacy. Many people with intellectual disabilities are on antipsychotic medication over years and sometimes decades [18, 26], despite associated health risks, including anticholinergic effects. However, following NHS England’s “Stopping over-medication of people with a learning disability, autism or both (STOMP)” initiative [15], there is some evidence of successful antipsychotic deprescribing [40], but these prescriptions may be being replaced with other psychotropics, namely, antidepressants [41], which could contribute to anticholinergic burden similarly. There is a need for robust evidence-based recommendations for all prescribers specifically about people with intellectual disabilities with multiple physical and mental ill-health conditions to optimise medication use. Although pharmacotherapy for those with intellectual disabilities is particularly complex, prescribers may not prioritise reducing the risk of anticholinergic burden. We report on the anticholinergic side effects; an important next research step will be to study the extent of anticholinergic burden in people with intellectual disabilities as measured by serum anticholinergic activity. Our results show that we should be cautious about the concurrent use of multiple low risk anticholinergic medications. It is imperative that anticholinergic prescribing, and particularly psychotropic prescribing, should be considered alongside the known health risks, and subsequently reviewed for efficacy, tolerability, and safety.

## Supplementary Information

**Additional file 1: Table S1. **Odds ratios for comparison of adults with intellectual disabilities and the general population adults for psychotropics with a significant interaction between group and age. **Table S2.** Odds ratios for comparison of adults with intellectual disabilities and the general population adults for antiepileptics by neighbourhood deprivation (SIMD quintile). **Figure S1. **Influence of neighbourhood deprivation (SIMD quintile) on groups’ MERs for predicted probability of being an antiepileptics.

## Data Availability

All data generated or analysed during the study are included in this published article (and its additional files).
