# Supplementary material for: A study on prescriptions contributing to the risk of high anticholinergic burden in adults with intellectual disabilities: retrospective record linkage study
Source: Ann Gen Psychiatry. 2022 Oct 29;21:41. doi: 10.1186/s12991-022-00418-x (PMC9617313; doi:10.1186/s12991-022-00418-x)

Additional Tables and Figures

Table 1. Odds ratios for comparison of adults with intellectual disabilities and the general population adults for psychotropics with a significant interaction between group and age.

| Model subpopulation | Number observations | Intellectual Disabilities Group Effect OR [95% CI], p-value | | |
| --- | --- | --- | --- | --- |
|  |  | Antipsychotics | Antidepressants | Anxiolytics/hypnotics |
| 17-24 | 134 | 2.16 [0.72-6.49], p=0.177 | 0.06 [0.01-0.27], p<0.001 | 2.81 [1.01-7.76], p=0.047 |
| 25-34 | 396 | 2.48 [1.34-4.60], p=0.004 | 0.56 [0.34-0.92], p=0.022 | 1.53 [0.84-2.79], p=0.168 |
| 35-44 | 492 | 2.44 [1.48-4.02, p<0.001 | 0.50 [0.29-0.84], p=0.009 | 0.90 [0.54-1.50], p=0.699 |
| 45-54 | 1,144 | 3.12 [2.24-4.34], p<0.001 | 0.76 [0.54-1.08], p=0.129 | 0.79 [0.55-1.14], p=0.206 |
| 55-64 | 1,225 | 9.07 [6.19-13.30], p<0.001 | 1.39 [0.93-2.06], p=0.105 | 2.01 [1.31-3.09], p=0.001 |
| 65-74 | 880 | 18.31 [9.12-36.78], p<0.001 | 2.21 [1.35-3.64], p=0.002 | 1.48 [0.84-2.59], p=0.172 |
| 75+ | 430 | 61.07 [13.11-284.44], p<0.001 | 1.94 [0.82-4.60], p=0.133 | 1.83 [0.98-3.43], p=0.060 |

OR=Odds Ratio, 95% CI=Confidence Interval and corresponding p-value for the logistic regression models for the effect of Intellectual Disabilities group compared to General Population group on each psychotropic, within each age subpopulation.

Table 2. Odds ratios for comparison of adults with intellectual disabilities and the general population adults for antiepileptics by neighbourhood deprivation (SIMD quintile).

| Model subpopulation | Number observations | Intellectual Disabilities Group Effect  OR [95% CI], p-value* |
| --- | --- | --- |
| SIMD 1-most deprived | 2,688 | 1.94 [1.59-2.37], p<0.001 |
| SIMD 2 | 936 | 2.54 [1.81-3.57], p<0.001 |
| SIMD 3 | 568 | 5.82 [3.69-9.18], p<0.001 |
| SIMD 4 | 308 | 6.98 [3.07-15.86], p<0.001 |
| SIMD 5-least deprived | 201 | 8.77 [3.64-21.15], p<0.001 |

*OR=Odds Ratio, 95% CI=Confidence Interval and corresponding p-value for the logistic regression models for the effect of Intellectual Disabilities group compared to General Population group on antiepileptics, within each SIMD subpopulation.

Supplementary Figure 1. Influence of neighbourhood deprivation (SIMD quintile) on groups’ MERs for predicted probability of being an antiepileptics.


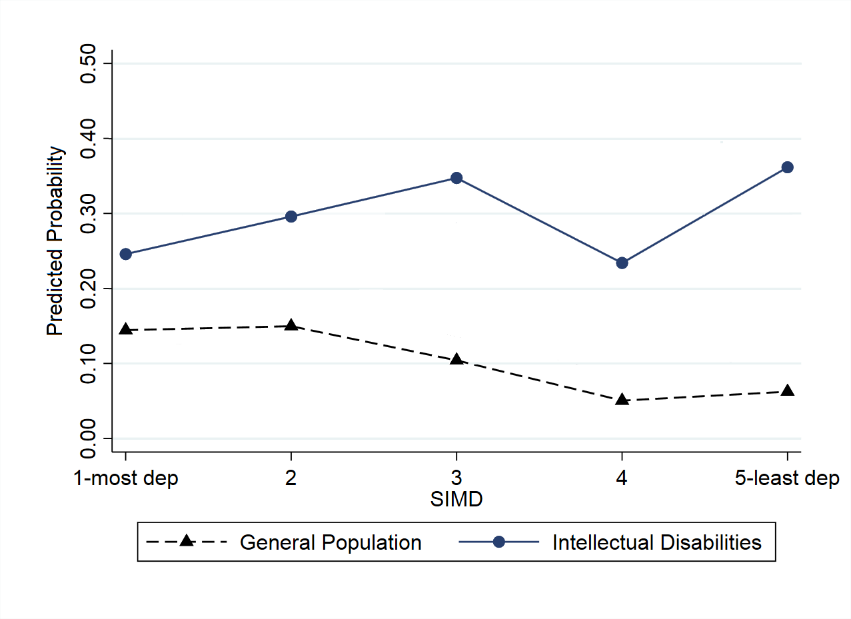

Supplement: Supplementary file 1 — Additional file 1: Table S1. Odds ratios for comparison of adults with intellectual disabilities and the general population adults for psychotropics with a significant interaction between group and age. Table S2. Odds ratios for comparison of adults with intellectual disabilities and the general population adults for antiepileptics by neighbourhood deprivation (SIMD quintile). Figure S1. Influence of neighbourhood deprivation (SIMD quintile) on groups’ MERs for predicted probability of being an antiepileptics. [file 12991_2022_418_MOESM1_ESM.docx]
